# CD147 and cyclophilin A: a promising potential targeted therapy for COVID-19 and associated cancer progression and chemo-resistance

**DOI:** 10.1186/s13027-023-00501-2

**Published:** 2023-04-04

**Authors:** Maryam Bakhtiyari, Ayda Haji Aghasi, Sara Banihashemi, Arian Abbassioun, Chanour Tavakol, Hamidreza Zalpoor

**Affiliations:** 1 Department of Medical Laboratory Sciences, Faculty of Allied Medicine, Qazvin University of Medical Sciences, Qazvin, Iran; 2 Network of Immunity in Infection, Malignancy & Autoimmunity (NIIMA), Universal Scientific Education & Research Network (USERN), Tehran, Iran; 3 Student Research Committee, Lorestan University of Medical Sciences, Khorramabad, Iran; 4 Diagnostic Laboratory Sciences and Technology Research Center, School of Paramedical Sciences, Shiraz University of Medical Sciences, Shiraz, Iran; 5 Department of Virology, Faculty of Veterinary Medicine, University of Tehran, Tehran, Iran; 6 School of Medicine, Tehran University of Medical Sciences, Tehran, Iran; 7 Shiraz Neuroscience Research Center, Shiraz University of Medical Sciences, Shiraz, Iran

**Keywords:** COVID-19, SARS-CoV-2, CD147, Cyclophilin A, Cancer progression, Chemo-resistance

## Abstract

Coronavirus disease-2019 (COVID-19), as a worldwide serious issue has been shown to lead to progression and poor outcomes in cancer patients. The underlying mechanisms for SARS-CoV-2 infection’s adverse effects on cancer patients have not been fully understood. We hypothesized that CD147 and Cyclophilin A (CyPA) not only can play a significant role in infection severity but also can contribute to cancer progression and chemotherapy resistance in cancer patients with COVID-19. In addition, we hypothesized that the expression of both CD147 and CyPA could be increased by Hypoxia-inducible Factor-1 alpha (HIF-1α) activation during hypoxic conditions that occurred during COVID-19. Therefore, this evidence can open a new window in the management of cancer patients during the pandemic and therapeutic approaches targeting CD147 and CyPA could be a potentially promising therapeutic approach for such patients.

Dear Editor,

Nowadays, a number of studies suggest that patients with primary diseases such as multiple cancers are more susceptible to severe Coronavirus disease-2019 (COVID-19) and mortality [1–3]. Severe acute respiratory syndrome coronavirus 2 (SARS-CoV-2) is responsible for COVID-19 infection and has a variety of cell and organ targets [4]. Studies revealed that SARS-CoV-2 attaches to host cells through binding its spike (S) protein to its specific receptors such as Angiotensin-Converting Enzyme 2 (ACE2), CD147, Ephrin receptors, Neuropilin-1, etc. [5–9]. In previous studies, we suggested that SARS-CoV-2 can stimulate downstream signaling pathways of these receptors, facilitating COVID-19-associated pathological complications and cancer progression [5, 6, 10–14].

CD147 is classified as a transmembrane glycoprotein and is a part of the immunoglobulin superfamily. CD147 can be found in several organs and cells including T-cells, endothelial cells, and multiple central nervous system (CNS) cells. Investigations found that CD147 plays a significant role in viral infections, particularly in SARS-CoV-2 infection [5]. CD147 interacts with the SARS-CoV-2-S protein and is able to penetrate host cells through binding to CD147 [7].

In the Cyclophilin family, Cyclophilin A (CyPA) is the most abundant protein. CyPA exerts a significant role in intracellular protein synthesis, transportation, and folding, along with signal transduction, and immunosuppression. Additionally, CyPA plays a key role in viral infections, including human immunodeficiency virus (HIV), hepatitis C virus (HCV), and hepatitis B virus (HBV). Recent studies suggest that CyPA/CD147 interaction may facilitate SARS-CoV-2 viral entry and replication (Fig. 1) [15]. Jiejie Geng et al. study revealed that CyPA expression was upregulated upon SARS-CoV-2 infection [16]. It has been revealed that due to hypoxic conditions, both CD147 and CyPA expression increases by Hypoxia-inducible factor 1-alpha (HIF-1α) [17, 18]. We suggest that HIF-1α activated by the hypoxic conditions that occur by COVID-19 infection can increase the expression of CD147 and CyPA in infected cells and exposed cells to the hypoxic conditions (Fig. 1). However, it has not been assessed whether CD147 elevates by SARS-CoV-2 or not. It has been demonstrated that the transcriptional activity of HIF-1α leads to CD147 expression. Based on this, HIF-1α activated by SARS-CoV-2 could elevate the expression of CD147 and this hypothesis can encourage researchers to consider it in future studies, opening a new window to find a new therapeutic approach for cancer patients with COVID-19.

**Fig. 1:**
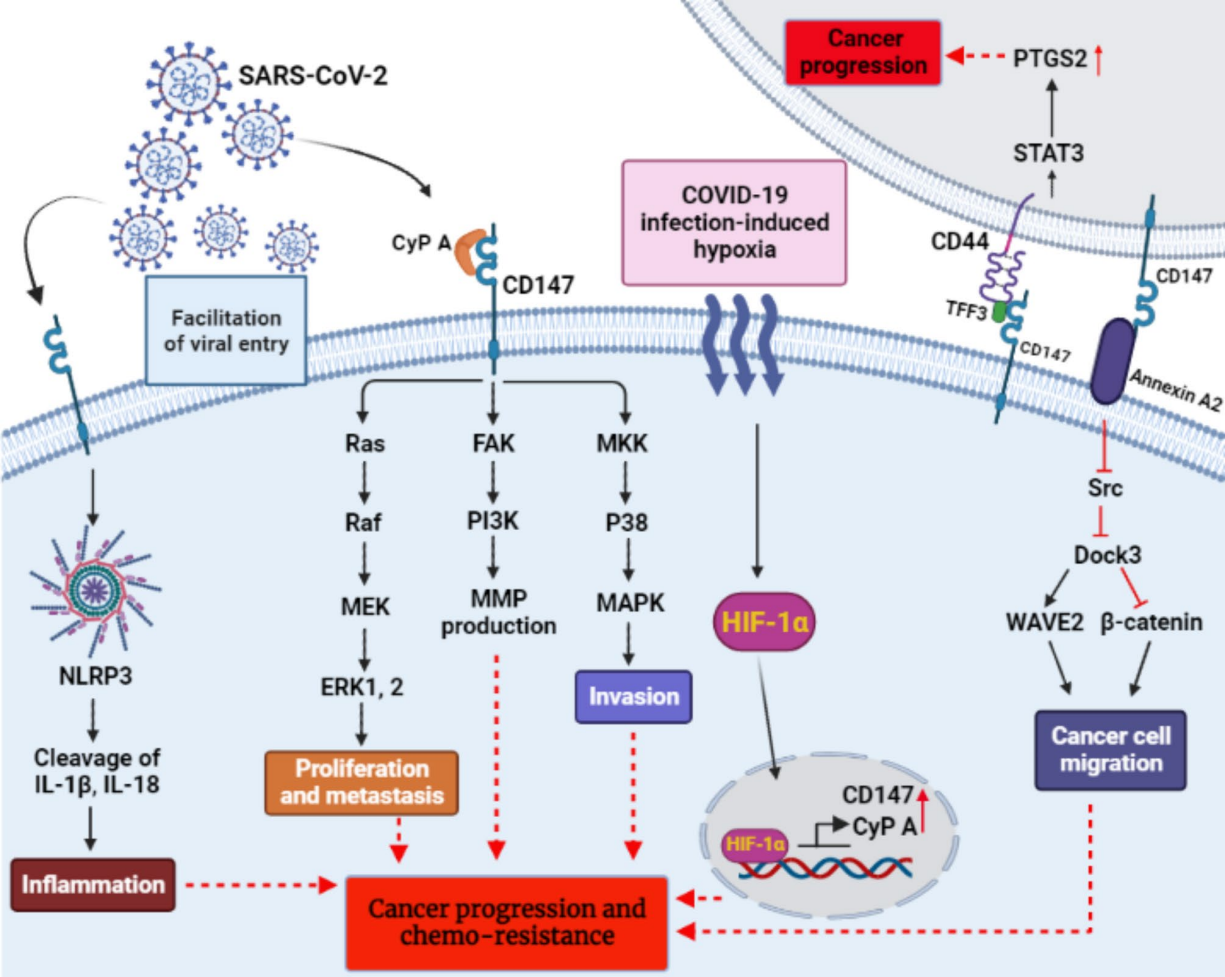
The illustration shows the role of CD147 as a SARS-CoV-2 entry receptor. SARS-CoV-2 can stimulate intracellular signaling pathways of CD147 in host cells (cancer cells). Also, COVID-19 infection-induced hypoxia can lead to HIF-1α activation and transcriptional activity to increase the expression of CD147 and CyPA. All of these can potentially contribute to cancer progression and chemo-resistance

Here, we hypothesized that CD147 and CyPA not only can contribute to SARS-CoV-2 infection by facilitating viral entry and replication but also can contribute to COVID-19-associated cancer progression and chemo-resistance.

Multiple pathways can potentially be initiated in the host cells, including mitogen-activated protein kinase (MAPK), P38, extracellular signal-regulated kinase (ERK) 1/2, and Phosphoinositide 3-kinase (PI3K), and NF-kappa B (NF-κB). Accordingly, as a consequence of CD147 stimulation, ERK phosphorylation, IKß phosphorylation-associated degradation, nuclear translation of NF-κB, and P65 subunits are induced. CD147 is recognized as an initiator of an inflammatory response in many cells such as macrophages. This can result in the induction of MMP-9 expression as well as the release of pro-inflammatory cytokines and cytokine storm [3].

Additionally, it has been suggested that CD147 as an entry receptor for SARS-CoV-2 enables the virus to penetrate the infected cell cytoplasm and activate the NLR family pyrin domain containing 3 (NLRP3) inflammasome that cleaves IL-1ß and IL-18 in COVID-19 patients [5, 19, 20].

According to many studies, CD147 can cause the progression of various cancers, including hematological malignancies and solid tumors. This receptor exerts its roles through various mechanisms [18]. For example, CD147 is a receptor for CyPA that causes metastasis through the ERK1/2 signaling pathway. This receptor also induces matrix metalloproteinases (MMPs) synthesis and cancer progression through the (FAK)-PI3K signaling pathway [5].

In pancreatic cancer tissues, CyPA and CD147 are expressed at higher levels. Pancreatic cancer cell proliferation can be significantly inhibited by the CD147 antibody [15]. Accordingly, the downstream signaling pathways Ras/Raf/MEK/ERK1/2 and MKK/p38/MAPK cause proliferation and invasion, respectively, and in general cause cancer progression [21].

In another report by Obchoei et al. it has been found that CypA expression is increased in cholangiocarcinoma and HIF-1α is one of the factors that lead to an increase in CypA expression. Increased CypA production in the cell can affect the CD147 receptor on the cell surface and trigger the ERK1/2 signaling pathway, leading to cancer progression (Fig. 1) [22].

In addition, according to H-Y Cui et al. studies, CD147 binds to CD44 (as an adhesion molecule and anti-apoptotic molecule) via TFF-3 and activates signal transducer and activator of transcription 3 (STAT3). STAT3 activation leads to an increase in PTGS2 and the progression of colorectal cancer (Fig. 1) [23].

Accordingly, it has been shown that the I domain of CD147 binds to the Annexin A2 N-terminal to prevent its phosphorylation with Src. This inhibition of phosphorylation impairs Dock3 expression, and since Dock3 inhibits β-catenin and induces WAVE2 expression (WAVE2 inhibits cell movement), the lack of Dock3 expression causes cancer migration [24].

In general, PI3K/AKT and MAPK are among the CD147 tumor progression signaling pathways that result in cytoskeleton movement (Fig. 1). In addition, vascular endothelial growth factor (VEGF) production in endothelial cells, facilitating glycolysis, proliferation, invasion, and inhibition of apoptosis in hypoxic conditions are also CD147 oncogenic functions. On the other hand, epididymis protein 4 (HE4) as a tumor marker has a significant increase in ovarian serous carcinoma compared to other cancers and causes the occurrence and development of ovarian carcinoma. According to research by L. Gao et al. Interaction of HE4 and CD147 in ovarian carcinoma can lead to the promotion of invasion and metastasis of ovarian carcinoma by forming a protein complex in which Annexin A2 acts as a bridge [25].

Studies have shown that cells with high expression of CD147 have an enhanced tumorigenic potential and chemo-resistance in-vivo [26]. High CD147 expression in glioma cells can cause temozolomide (TMZ) resistance and in ovarian cancer, tissues can cause paclitaxel resistance [27, 28]. In glioma cells, suppression of CD147 expression increased the inhibitory effect of TMZ on cell survival in U251 and T98G when increased CD147 function blocked reactive oxygen species (ROS) production and apoptosis from TMZ [28]. Expression of nuclear factor E2-related factor 2 (Nrf2) which depends on CD147 can contribute to the reduction of ROS production consequences of TMZ. CD147 could establish the Nrf2 constant by suppression of GSK3β/β-TrCP Dependent protein [28]. Cells with high levels of CD147 expression displayed an increased expression of EGF receptors (EGFR), ABCG1, ABCG2 drug transporters, and MCT4 monocarboxylate transporters [26]. Increasing expression of CD147 and γ lewis antigen was observed in ovarian epithelium with chemo-resistant properties [29]. It has been shown that cisplatin-resistant ovarian cancers express high levels of MD1/P-gp and CD147/CD98hc complexes. Lewis γ antigen as a part of the CD147 structure could affect chemo-resistance in ovarian cancer by stimulating the ERK1/2 signaling pathway. Hence, CD147 could be an important index for clinical purposes and prognosis [29].

Studies have demonstrated that chemo-resistant colorectal cancer (CRC) samples have higher levels of CypA, which predicts a poor prognosis in these patients. Additionally, cyclosporine A, by targeting CypA, exhibits promising efficacy against chemo-resistant CRC when combined with chemotherapy [30].

Studies have found that CyPA inhibitors-alisporivir can inhibit the replication of the SARS-CoVs and the Middle East respiratory syndrome (MERS), and ribavirin enhances the anti-viral activity of alisporivir [15].

In conclusion, this evidence suggests that CD147 and CyPA are not only potential therapeutic targets for COVID-19 but can also be beneficial for cancer patients with COVID-19 to hinder cancer progression and chemo-resistance. Also, we hypothesized that the expression of CD147 and CyPA could be increased by HIF-1α activated by COVID-19, suggesting risk factors for long-term COVID-19 complications for cancer patients. This is a hypothesis that tumor cells could be affected by hypoxic condition and HIF-1α stimulation by SARS-CoV-2 infection like other infected cells. However, due to lack of evidence, it encourages researchers that consider it in future investigations to evaluate this suggestion. In future studies, this study may shed light on developing novel therapeutic approaches for cancer patients with COVID-19 to reduce mortality and treatment failure.

## Data Availability

All data presented in this article are totally available and present in the text.

## References

[1] Nabi-Afjadi M, Mohebi F, Zalpoor H, Aziziyan F, Akbari A, Moradi-Sardareh H et al. A cellular and molecular biology-based update for ivermectin against COVID-19: is it effective or non-effective? Inflammopharmacology. 2023:1–15. 10.1007/s10787-022-01129-1 PMC9823263 36609716

[2] Samidoust P, Delshad ME, Talemi RN, Mojtahedi K, Samidoust A, Jahangiri S et al. Incidence, characteristics, and outcome of COVID-19 in patients on liver transplant program: a retrospective study in the north of Iran. New Microbes New Infect. 2021;44:100935. 10.1016/j.nmni.2021.100935 PMC8413100 34493955

[3] Aghajanzadeh M, Haghighi M, Rimaz S, Fomani AA, Tangestaninejad A, Ashoobi MT (2021). Pneumomediastinum, pneumopericardium pneumothorax and subcutaneous emphysema in iranian COVID-19 patients. J Curr Biomedical Rep.

[4] Zalpoor H, Akbari A, Nabi-Afjadi M, Forghaniesfidvajani R, Tavakol C, Barzegar Z et al. Hypoxia-inducible factor 1 alpha (HIF‐1α) stimulated and P2X7 receptor activated by COVID-19, as a potential therapeutic target and risk factor for epilepsy. Human Cell. 2022:1–8. 10.1007/s13577-022-00747-9 PMC9281298 35831562

[5] Zalpoor H, Akbari A, Samei A, Forghaniesfidvajani R, Kamali M, Afzalnia A (2022). The roles of eph receptors, neuropilin-1, P2 × 7, and CD147 in COVID-19-associated neurodegenerative diseases: inflammasome and JaK inhibitors as potential promising therapies. Cell Mol Biol Lett.

[6] Zalpoor H, Akbari A, Nabi-Afjadi M. Ephrin (Eph) receptor and downstream signaling pathways: a promising potential targeted therapy for COVID–19 and associated cancers and diseases.Human Cell. 2022:1–3. 10.1007/s13577-022-00697-2 PMC8977187 35377105

[7] Wang K, Chen W, Zhang Z, Deng Y, Lian J-Q, Du P (2020). CD147-spike protein is a novel route for SARS-CoV-2 infection to host cells. Signal Transduct Target therapy.

[8] Beaudoin CA, Jamasb AR, Alsulami AF, Copoiu L, van Tonder AJ, Hala S (2021). Predicted structural mimicry of spike receptor-binding motifs from highly pathogenic human coronaviruses. Comput Struct Biotechnol J.

[9] Zalpoor H, Liaghat M, Bakhtiyari M, Shapourian H, Akbari A, Shahveh S (2023). Kaempferol’s potential effects against SARS-CoV-2 and COVID-19-associated cancer progression and chemo-resistance.

[10] Zalpoor H, Bakhtiyari M, Liaghat M, Nabi-Afjadi M, Ganjalikhani‐Hakemi M. Quercetin potential effects against SARS‐CoV‐2 infection and COVID‐19‐associated cancer progression by inhibiting mTOR and hypoxia‐inducible factor‐1α (HIF‐1α).Phytotherapy Research. 2022. 10.1002/ptr.7440 PMC9111038 35307904

[11] Zalpoor H, Bakhtiyari M, Shapourian H, Rostampour P, Tavakol C, Nabi-Afjadi M. Hesperetin as an anti-SARS-CoV-2 agent can inhibit COVID-19-associated cancer progression by suppressing intracellular signaling pathways.Inflammopharmacology. 2022:1–7. 10.1007/s10787-022-01054-3 PMC9393098 35994216

[12] Zalpoor H, Shapourian H, Akbari A, Shahveh S, Haghshenas L. Increased neuropilin-1 expression by COVID-19: a possible cause of long-term neurological complications and progression of primary brain tumors.Human Cell. 2022:1–3. 10.1007/s13577-022-00716-2 PMC9084541 35534753

[13] Zalpoor H, Rezaei M, Yahyazadeh S, Ganjalikhani-Hakemi M. Flt3-ITD mutated acute myeloid leukemia patients and COVID-19: potential roles of autophagy and HIF-1α in leukemia progression and mortality.Human Cell. 2022:1–2. 10.1007/s13577-022-00718-0 PMC9152658 35639283

[14] Zalpoor H, Akbari A, Nayerain Jazi N, Liaghat M, Bakhtiyari M (2022). Possible role of autophagy induced by COVID-19 in cancer progression, chemo-resistance, and tumor recurrence. Infect Agents Cancer.

[15] Liu C, von Brunn A, Zhu D, Cyclophilin (2020). A and CD147: novel therapeutic targets for the treatment of COVID-19. Med drug discovery.

[16] Geng J, Chen L, Yuan Y, Wang K, Wang Y, Qin C (2021). CD147 antibody specifically and effectively inhibits infection and cytokine storm of SARS-CoV-2 and its variants delta, alpha, beta, and gamma. Sig Transduct Target Ther..

[17] Zhang H, Chen J, Liu F, Gao C, Wang X, Zhao T (2014). CypA, a gene downstream of HIF-1α, promotes the development of PDAC. PLoS ONE.

[18] Lian C, Guo Y, Zhang J, Chen X, Peng C (2017). Targeting CD147 is a novel strategy for antitumor therapy. Curr Pharm Design.

[19] Chen I-Y, Moriyama M, Chang M-F, Ichinohe T (2019). Severe acute respiratory syndrome coronavirus viroporin 3a activates the NLRP3 inflammasome. Front Microbiol.

[20] Merad M, Martin JC (2020). Pathological inflammation in patients with COVID-19: a key role for monocytes and macrophages. Nat Rev Immunol.

[21] Xin X, Zeng X, Gu H, Li M, Tan H, Jin Z (2016). CD147/EMMPRIN overexpression and prognosis in cancer: a systematic review and meta-analysis. Sci Rep.

[22] Obchoei S, Weakley SM, Wongkham S, Wongkham C, Sawanyawisuth K, Yao Q (2011). Cyclophilin a enhances cell proliferation and tumor growth of liver fluke-associated cholangiocarcinoma. Mol Cancer.

[23] Cui H-Y, Wang S-J, Song F, Cheng X, Nan G, Zhao Y (2021). CD147 receptor is essential for TFF3-mediated signaling regulating colorectal cancer progression. Sig Transduct Target Ther..

[24] Cui H-Y, Wang S-J, Miao J-Y, Fu Z-G, Feng F, Wu J (2016). CD147 regulates cancer migration via direct interaction with annexin A2 and DOCK3-β-catenin-WAVE2 signaling. Oncotarget.

[25] Gao L, Nie X, Gou R, Qi Y, Liu J, Lin B (2021). Interaction of CD147 and human epididymis protein 4 promotes invasion and metastasis of ovarian cancer. J Cancer.

[26] Dai L, Guinea MC, Slomiany MG, Bratoeva M, Grass GD, Tolliver LB (2013). CD147-dependent heterogeneity in malignant and chemoresistant properties of cancer cells. Am J Pathol.

[27] Nan G, Zhao S-H, Wang T, Chao D, Tian R-F, Wang W-J et al. CD147 supports paclitaxel resistance via interacting with RanBP1.Oncogene. 2022:1–14. 10.1038/s41388-021-02143-3 PMC8837534 34974521

[28] Bu X, Qu X, Guo K, Meng X, Yang X, Huang Q (2021). CD147 confers temozolomide resistance of glioma cells via the regulation of β-TrCP/Nrf2 pathway. Int J Biol Sci.

[29] Gao J, Hu Z, Liu J, Liu D, Wang Y, Cai M (2014). Expression of CD147 and Lewis y antigen in ovarian cancer and their relationship to drug resistance. Med Oncol.

[30] Peng L, Jiang J, Chen H-N, Zhou L, Huang Z, Qin S (2021). Redox-sensitive cyclophilin a elicits chemoresistance through realigning cellular oxidative status in colorectal cancer. Cell Rep.

